# Habitual sleep is associated with both source memory and hippocampal subfield volume during early childhood

**DOI:** 10.1038/s41598-020-72231-z

**Published:** 2020-09-17

**Authors:** Tracy Riggins, Rebecca M. C. Spencer

**Affiliations:** 1 Department of Psychology, University of Maryland, 4094 Campus Dr, College Park, MD 20742 USA; 2 Department of Psychological and Brain Sciences, University of Massachusetts, 135 Hicks Way, Amherst, 01003 USA; 3 Institute for Applied Life Sciences, University of Massachusetts, 240 Thatcher Way, Amherst, 01003 USA

**Keywords:** Hippocampus, Long-term memory

## Abstract

Previous research has established important developmental changes in sleep and memory during early childhood. These changes have been linked separately to brain development, yet few studies have explored their interrelations during this developmental period. The goal of this report was to explore these associations in 200 (100 female) typically developing 4- to 8-year-old children. We examined whether habitual sleep patterns (24-h sleep duration, nap status) were related to children’s performance on a source memory task and hippocampal subfield volumes. Results revealed that, across all participants, after controlling for age, habitual sleep duration was positively related to source memory performance. In addition, in younger (4–6 years, n = 67), but not older (6–8 years, n = 70) children, habitual sleep duration was related to hippocampal head subfield volume (CA2-4/DG). Moreover, within younger children, volume of hippocampal subfields varied as a function of nap status; children who were still napping (n = 28) had larger CA1 volumes in the body compared to children who had transitioned out of napping (n = 39). Together, these findings are consistent with the hypothesis that habitually napping children may have more immature cognitive networks, as indexed by hippocampal integrity. Furthermore, these results shed additional light on why sleep is important during early childhood, a period of substantial brain development.

## Introduction

Early childhood is a period of dramatic development across multiple domains. For example, sleep shows dramatic changes as children transition from a biphasic sleep pattern (a nap and overnight sleep bout) to a monophasic sleep pattern (overnight sleep only)^1^. Sleep duration also changes between 3 and 7 years of age. Mean sleep duration drops from 12.5 h to 10.5 h from 3 to 7 years of age, then it becomes relatively stable into adolescence^2^. Concurrently, memory also shows accelerated change. The ability to recall autobiographical events increases rapidly between 3 and 7 years of age^3,4^. Children’s ability to recall details of laboratory-based tasks also shows accelerated changes during this period e.g.,^5^. It is likely that brain maturation contributes to these (and other) developmental changes. However, variations in sleep, memory and brain development are often explored separately and thus their interdependence remains unclear. In the present investigation we sought to explore these relations in an existing dataset of 200 children spanning early to middle childhood (4–8 years).

Sleep contributes to performance on memory tasks. In young adults, memory is greater following a period of sleep compared to a period of wake of the same duration (for reviews see^6–8^). This sleep benefit on memory is thought to reflect memory consolidation. Memories, initially supported by the hippocampus, are stabilized in the cortex. Synchronous hippocampal ripples, slow waves, and spindle bursts across the cortex are thought to provide a mechanisms for memory consolidation^9^.

Sleep in early childhood has likewise been shown to contribute to successful memory performance. Such benefits have been observed on declarative memory tasks^10–13^, emotional memory tasks^14^, and procedural memory tasks^15^. Studies examining the acute effects of a single sleep bout (e.g., an afternoon nap) on children’s memory performance find little evidence that the duration of the sleep period is predictive of improvements in memory^10,16^. Rather, results suggest that sleep physiology (e.g., sleep slow waves and spindles) is a better predicter of memory changes over the interval of sleep^10,14,15^. However, these acute effects may add up over time, as longer *habitual* sleep duration (i.e., the average amount of sleep a child gets per day) has been associated with better measures of global cognitive function^17,18^, including memory^19^.

Naps are sufficient for memory consolidation. For instance, when 3- to 5-year-old children were taught a visuo-spatial task (like the game ‘Memory’) in the morning, then assessed on their performance following an afternoon nap, memories were protected. However, when children were kept awake during the afternoon nap interval, memory was reduced^10^. Changes in memory performance over the nap were associated with sleep physiology, specifically sleep spindles. There was no association with age and only a small relation with nap duration. Moreover, when nap benefits were considered separately for children who napped habitually (5 or more naps/week) and those who napped non-habitually (0–1 nap/week), the benefit of the nap was found to be equal for both groups; naps preserved memories regardless of nap habituality. What differed between the two groups was the wake condition; when kept awake during nap time, memory decay was greater for children who napped habitually than for non-habitually napping children^10^. This is consistent with a study of habitual napping and memory relationships. Lam et al.^20^ observed a negative relation between nap frequency (akin to habitual and non-habitual nappers) and children’s global cognitive function. Specifically, fewer naps were associated with greater vocabulary and better memory for digit sequence, over and above the effects of age.

Together these findings suggest that children who have transitioned out of napping, regardless of age, may have sufficient cognitive resources that enable them to hold onto memories throughout the day in spite of interference from ongoing activities during wake. Conversely, habitually napping children may have less robust cognitive resources (perhaps due to lower hippocampal volume or integrity), which may explain why memories are more susceptible to interference when such children are kept awake^10,20^.

The brain undergoes significant development during early childhood, see^21^. Notably, this protracted development includes regions important for successful memory performance, including the hippocampus. Although early studies did not report dramatic differences in total hippocampal volume across development, e.g.,^22^, subsequent studies revealed age-related variation in subdivisions of the hippocampus. These divisions include subregions or divisions along the longitudinal axis (i.e., head, body, tail^23^) and subfields, which are cytoarchitecturally distinct regions that make up the major signaling pathways within the structure^24–26^. Hippocampal subfields include the dentate gyrus (DG), cornu ammonis areas 1–4 (CA1–4), and the subiculum. Research in non-human primates suggests DG and CA3 as having the most protracted developmental course, with notable maturation occurring until 7 years of age^25^. Research in humans supports this assertion and extends it to include prolonged development of CA1 as well, but neither animal nor human data suggest changes in subiculum^26,27^. These age-related differences include both increases e.g.,^26^ and decreases, e.g.,^28^ in volume, depending on the age group and subregion (head or body) of the hippocampus investigated. Differences in volume may reflect numerous changes at the cellular level, including variations in the number or size of neurons or alterations in synaptic connections (enhancement and/or pruning^29^).

**Figure 1:**
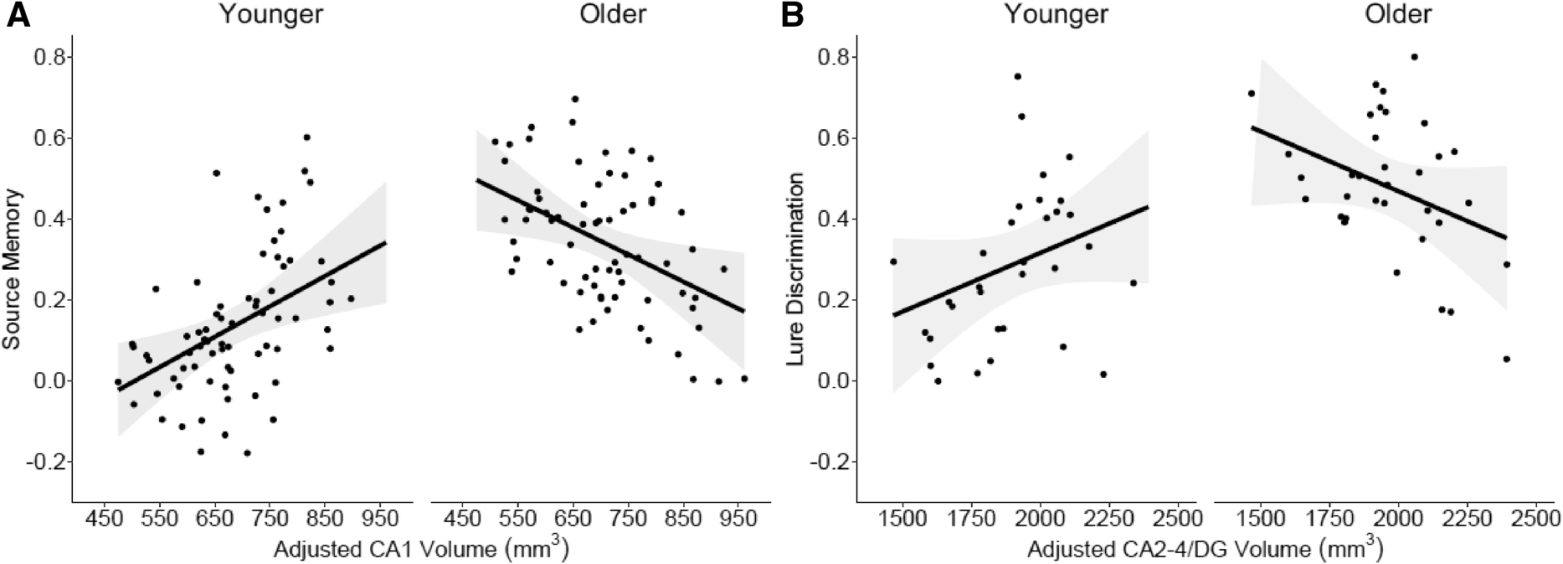
(**A**) Relations between source memory performance and CA1 volume in the head of the hippocampus for younger (4–6 year old; left) and older (6–8 year old; right) children (adapted from Riggins et al.^26^). (B) Relations between performance on a mnemonic similarity task and CA2–4/DG volume in the hippocampus for younger (left) and older (right) children (adapted from Canada et al.^24^).

Regardless of the physiological source of the variation, differences in volume of hippocampal subfields have been related to memory development, particularly during early childhood, even after controlling for effects of age and sex. Specifically, hippocampal subfield volumes have been associated with performance on a source memory task (CA1^26^) and a mnemonic discrimination task (CA2–4/DG^24^). In both studies, these relations were moderated by age: in younger children (~ 4 to 6 years), *larger* hippocampal subfield volumes were related to better memory performance, but the opposite was true in older children (~ 6 to 8 years), *smaller* hippocampal subfield volumes were related to better memory performance (Fig. 1).

Given the role of sleep in memory stabilization, we considered whether habitual sleep patterns may serve as a mechanism for variations in memory and brain development. Specifically, we considered whether 24-h sleep duration and nap status were related to memory and hippocampal subfield volumes in a sample of 4- to 8- year-old children. We hypothesized that habitual sleep would predict performance on a source memory task. Moreover, given that relations between hippocampal subfields and memory vary with age^24,26,30^, we predicted that greater habitual sleep would be associated with greater hippocampal subfield volume in younger but not older children. Finally, we also predicted that nap status would relate to both memory performance and hippocampal subfield volume.

## Results

**Figure 2:**
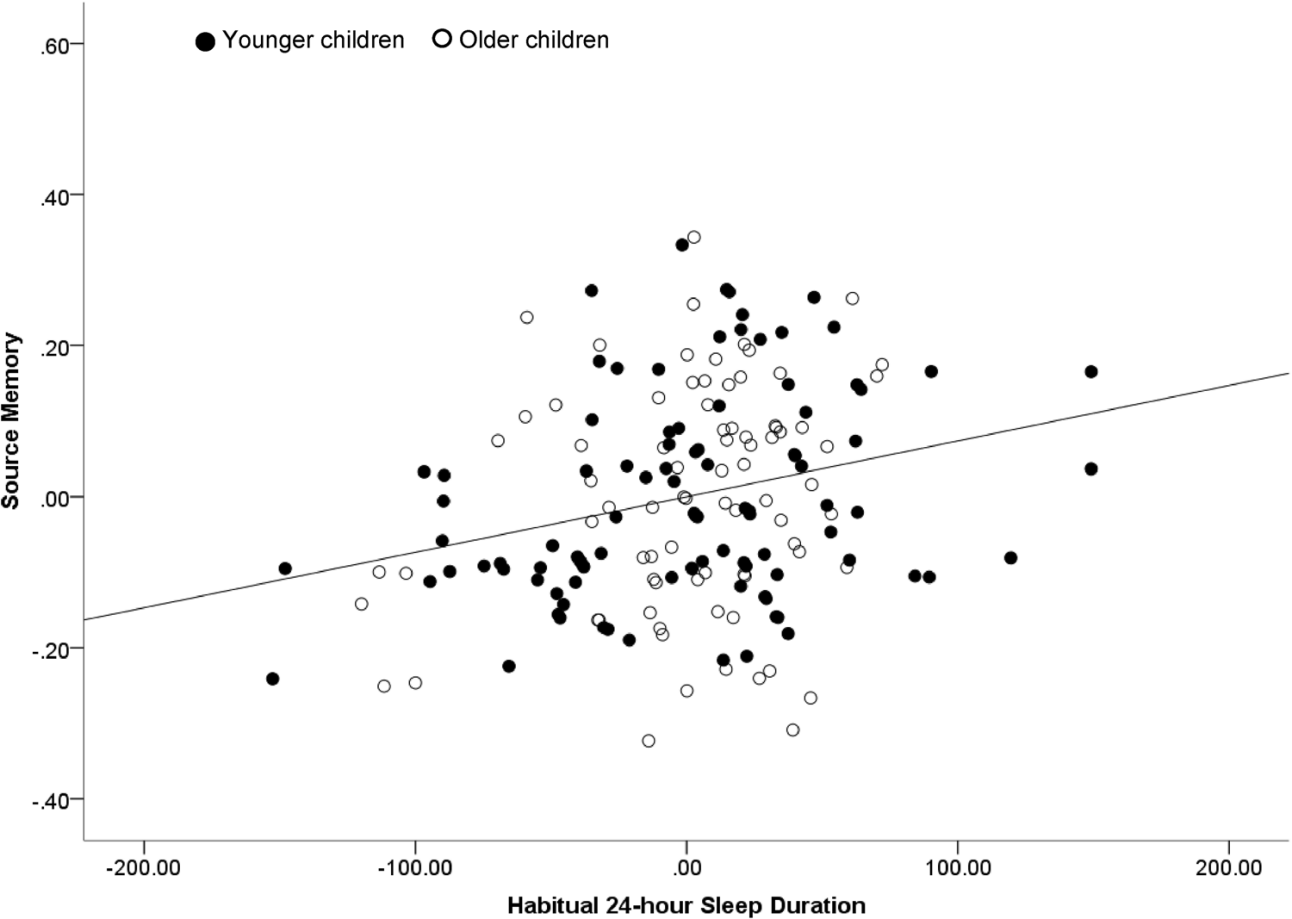
Partial regression plot illustrating the relation between habitual 24-h sleep duration and source memory, controlling for age and sex (n = 180). Age grouping were determined via median split.

Results revealed that, after controlling for age and sex, habitual 24-h sleep duration was related to performance on the source memory task across the whole sample, r(176) = 0.242, *p* = 0.001. Specifically, longer sleep durations were related to better ability to remember both the fact and its source after a 1-week delay (Fig. 2). This relation between habitual 24-h sleep duration and source memory performance held for both younger (r(98) = 0.294, *p* = 0.003) and older (r(74) = 0.229, *p* = 0.047) children based on a median split of age (6.04 years). The correlation in the younger children was not significantly greater than the correlation in the older children when tested using a Fisher r-to-z transformation, z = 0.46, *p* = 0.322, suggesting a similar association in both age groups.

**Figure 3:**
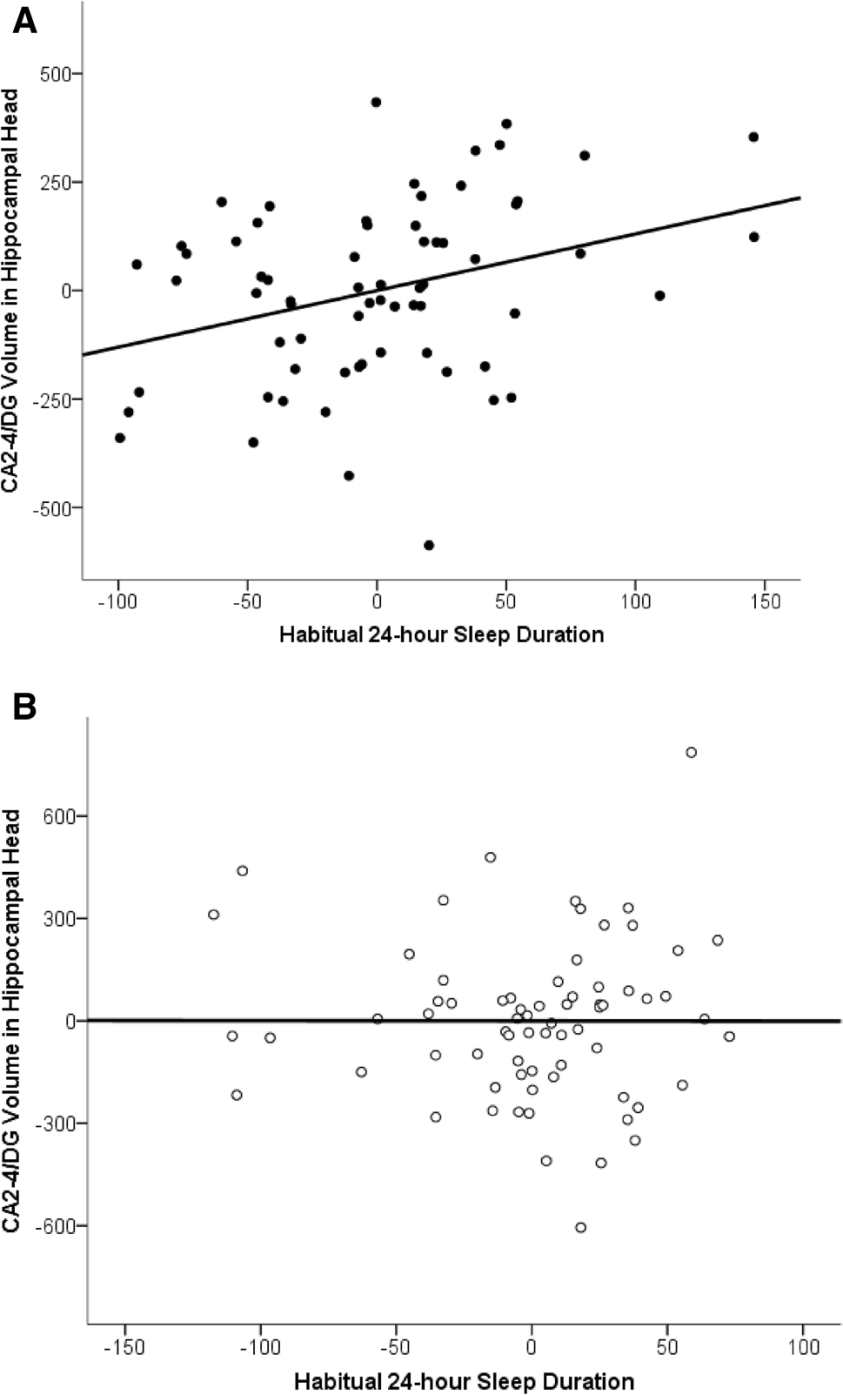
Partial regression plot illustrating relations between 24-sleep duration and ICV-adjusted hippocampal head subfield volumes CA2–4/DG in (**a**) younger (n = 67) and (**b**) older (n = 70) children, after controlling for age and sex.

Results also revealed that, after controlling for age and sex, habitual 24-h sleep duration was not related to ICV-adjusted volume of hippocampal subfields. However, when younger and older children were examined separately, significant relations emerged in younger but not older children. Specifically, in younger children, there was a positive association between habitual 24-h sleep duration and ICV-adjusted volume of CA2-4/DG in the head of the hippocampus, r(63) = 0.328, *p* = 0.008 (Fig. 3A). Longer sleep durations were associated with larger subfield volumes. There were no significant relations in older children, r(66) = − 0.001, *p* = 0.992 (Fig. 3B). The correlation in the younger children was significantly greater than the correlation in the older children when tested using a Fisher r-to-z transformation, z = 1.95, *p* = 0.026, suggesting different associations in the two age groups.

**Figure 4:**
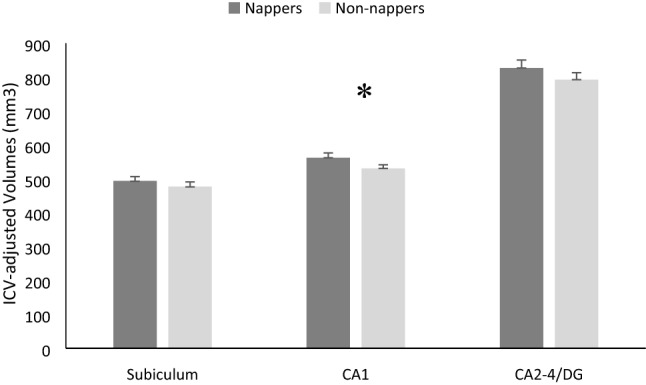
Mean ICV-adjusted volumes for subfields in the hippocampal body in younger nappers (n = 28) versus younger non-nappers (n = 39). *p < .05.

Finally, we examined whether nap status was related to memory or brain development, controlling for age and sex (Table 1). There were no differences in source memory as a function of nap status, *F*(1,97) = 3.389, *p* = 0.069. However, ICV-adjusted volumes of CA1 in the body of the hippocampus differed as a function of nap status within the younger age group, *F*(1,63) = 4.964, *p* = 0.029, ηp^2^ = 0.073. Volumes in nappers were larger than volumes in non-nappers (Fig. 4). In order to determine the specificity of these differences, we examined differences between young nappers and young non-nappers in 24-h sleep duration, estimates of verbal and spatial IQ, volume of the amygdala (a nearby structure) not hypothesized to differ as a function of nap status, and global brain metrics including total gray matter volume, subcortical gray matter volume, and ICV (Table 1). No differences were apparent in any of these variables.

**Table 1:** Descriptive statistics for nappers (n = 27–43) and non-nappers (n = 37–60) within the younger half of the sample.

	Nappers		Non-nappers		Group differences
	Mean	SD	Mean	SD	
Age (years)	4.619	.575	5.355	.721	*F*(1,65) = 16.203, *p* < .001
Source memory (% correct)	13.83%	15.55	15.95%	15.62	*ns*
24-h sleep (min)	631.500	69.107	615.000	45.964	*ns*
Block design (SS)	11.428	2.881	11.433	3.116	*ns*
Vocab (SS)	13.024	3.181	13.400	2.585	*ns*
Subcortical gray matter volume (mm^3^)	180,425.851	18,442.7305	187,647.162	12,121.734	*ns*
Total gray matter volume (mm^3^)	763,904.757	62,615.0560	775,710.237	51,612.3048	*ns*
Intracranial volume (mm^3^)	1,306,752.024	112,369.782	1,340,497.668	115,688.771	*ns*
Amygdala (mm^3^)	2,948.185	370.813	3,022.676	297.540	*ns*

Group differences in memory, sleep, estimates of IQ, and brain were examined using ANCOVAs, controlling for both age and sex.

*ns* not significant, *SS* scaled scores.

## Discussion

The present study documents relations between habitual sleep, memory, and hippocampal subfield volume in early childhood. Specifically, habitual 24-h sleep duration predicted source memory across the entire sample of 4- to 8-year-old children, after controlling for effects of age and sex. In addition, habitual 24-h sleep duration predicted volume of hippocampal head subfields CA2–4/DG. However, this finding was specific to younger children (4- to 6-years of age), suggesting timing-dependent associations may exist across childhood. Finally, within younger children, there were differences in hippocampal body subfield volumes between napping and non-napping children. Younger children who were still napping had larger CA1 subfield volumes in the body compared to non-napping children. Given some previous research showing age-related decreases in volume of hippocampal body subfields, e.g.,^28^, cf.^23^, these findings are consistent with the hypothesis that transitioning out of napping may be related to brain maturation, specifically, hippocampal development. It is notable that nap status was not related to volume of adjacent structures (i.e., the amygdala) nor global brain metrics (e.g., total gray matter volume). Overall, this set of findings is consistent with the proposal that habitually napping children have less robust cognitive resources due to immaturity of the hippocampus, and thus may require a nap mid-day to preserve memories over the long term, e.g.,^10^. These findings are important, as they shed light on why sleep is particularly important for memory, especially during early childhood, as sleep, memory, and the brain all show substantial developmental change.

These findings align well with previous work on relations between sleep and memory in childhood. Specifically, previous work has not only established relations between sleep duration and cognitive ability^17,19^, but also established how physiology during a specific bout of sleep promotes memory consolidation^10,14^. Our findings showing relations between habitual 24-h sleep duration and source memory performance across 4- to 8-year-old children extend the previous work to include a new declarative memory task that requires binding of details and retaining this association across a significant delay (i.e., 1 week). In addition, our findings suggest a neurobiological mechanism that may support this ability, particularly in younger children. Volumes of subfields have been shown previously to relate to performance on this source memory task^26^, as well as another memory task that requires memory for precise details (mnemonic similarity task, see Canada et al.^24^ for details). Hippocampal head subfield volumes (i.e., CA2–4/DG) showed relations with 24-h sleep duration in younger children in the present report. Finally, previous research on sleep physiology and memory consolidation across a nap suggests sleep spindles and slow oscillations play a role^10,14^ see^31^ for review. Memory consolidation during sleep is thought to arise from memory replay in the hippocampus, which is stabilized in the cortex through simultaneous sleep spindles and slow oscillations co-occurring across the cortex^9^, and may serve as the neurobiological mechanism underlying these reported relations.

Previous research has also suggested that the transition from biphasic to monophasic sleep may reflect brain development. Specifically, although naps are equally beneficial (memories encoded in the morning were protected) for habitual (≥ 5 naps/week) and non-habitual nappers (≤ 2 naps/week), preventing naps has differential effects^10^. Staying awake during a regular nap opportunity is detrimental for habitual nappers and results in significant forgetting. Staying awake for the same time does not impair memories for non-nappers of the same age. This finding suggested that the hippocampus of the non-habitual nappers matured to a point where it (and its associated network) could hold memories for the full day without catastrophic interference. For habitual nappers, this shorter-term memory storage may not have been sufficient to hold the memories from a full day, thus, memories had to be ‘downloaded’ (i.e., stabilized via increased cortical storage) more frequently via consolidation over the nap. Our findings provide empirical support for this proposal.

The finding that there were no significant differences in memory performance between the nappers and non-nappers was unexpected. However, the delay over which the information was to be retained was much greater (i.e., 1 week) in the present study compared to the previous study (< 1 day). It is possible that multiple bouts of overnight sleep could have contributed to memory performance in the present case. In the present study, smaller hippocampal subfield volumes were associated with nap transition (i.e., smaller volumes were observed in non-nappers compared to nappers). This finding is in line with previous research showing age-related decrease in hippocampal body subfield volumes, e.g.,^24,28^ and studies showing age-related variations in relations between memory and hippocampal subfield volumes. Specifically, in previous studies, smaller volumes have been associated with better memory performance in older children^24,26,30^, perhaps reflecting synaptic pruning (as suggested by^29^).

Although the present findings break new ground, there are a few limitations worth noting. First, the present findings were conducted between-subjects and thus do not give a clear account of how these changes may look within an individual child. Given the extent of variability between individuals in memory, sleep, and the brain, we cannot rule out the possibility that these effects reflect differences between groups of children as opposed to true developmental change. However, this initial work is exciting and lays the groundwork for future longitudinal investigations. Second, sleep was measured via parent report and thus may have contained errors. Parents tend to overestimate child sleep time^32^, and parents may not be well-informed of nap habituality for children who nap in preschool or daycare. However, such inaccuracies are likely global, affecting reports of young and older child and habitual and non-habitually napping children equally, and are unlikely to account for the present results. We recommend that future studies include objective sleep measures such as actigraphy. Finally, we would like to acknowledge the need for replication of these findings. Given the exploratory nature of this work and the moderate strength of the correlations and effect sizes, replication would significantly bolster the conclusions.

Despite these limitations, this study is the first to document relations between sleep, memory, and hippocampal subfield volumes in early childhood. These findings contribute to our understanding of possible mechanisms underlying change in early memory development. In addition, these initial findings have the potential to impact other fields, such as early education. Although quiet rest periods are currently routine in most preschools, they are often short (< 45 min), unstructured, and threatened by increasing curriculum-based learning. There are currently no guidelines regarding the promotion of naps or the appropriate length of the nap opportunity. If our hypotheses prove correct, naps could be seen as an asset to early education, and educational practices promoting naps during development should be considered for some children. Ultimately, we hope this study will encourage future research at intersection of child development, sleep, memory, and brain development, and that together we can begin to build scientifically-based guidelines and policies regarding sleep and napping during early childhood.

## Method

This study was approved by the Institutional Review Board (IRB) at the University of Maryland prior to any data collection. All research was performed in accordance with guidelines and regulations set forth by the IRB. All participants’ guardians provided informed consent, and all participants provided informed assent (written for ages 7 years and above).

### Participants

A total of 200 children (n = 100 female) ages 4–8 years were recruited from an urban area via a university-maintained database consisting of families willing to be contacted for research studies and from flyers posted near local schools. Inclusion criteria included: typical development, born at term, fluent in English, and no first-degree relatives with developmental disorders. A subset of these children were followed longitudinally for 3 years but are not included in the present report due to the fact that, by the second follow up wave, nearly all children had transitioned out of napping. Previous reports on structural and functional variations of the hippocampus in this sample include: Bauer et al.^33^, Canada et al. ^24^, Geng et al.^34^; Riggins et al.^26^. However, these publications were focused on age-related differences in hippocampal structure and function, and none evaluated the role of habitual sleep in these relations.

For the present report we examined relations between performance on a source memory task, estimates of verbal and spatial IQ, Child Sleep Habits Questionnaire (CSHQ^35,36^), and volume of hippocampal subfields in both the head and the body. Of the original sample, 184 participants accurately completed the CSHQ and 137 children provided useable volumetric MRI data.

### Measures

#### Source memory task, see ^26^

This task was adapted from^5,37^ in order to examine children’s ability to remember the details of a previous learning experience. Results of children’s performance on the source memory task from this sample have been reported elsewhere^26^. In short, in a laboratory setting, children were taught 12 novel facts (e.g., “A group of rhinos is called a crash”) from one of two different sources (puppet or person) via video. Presentation of facts was blocked by source, so that children learned 6 facts from the first source followed by 6 facts from the second source, and the order of the blocks was randomly assigned across participants. Children were told to pay attention to the facts as they would be tested on them the following week. However, children were not told that they would be tested on the source of the facts. After a 1-week delay, children returned to the lab and, in the context of a trivia game, were asked to answer 22 questions and to provide the source their answers (e.g. the puppet or the person). Five facts probed information commonly known by children (e.g., “What color is the sky?”), 5 facts probed information children typically would not know (e.g., “What is the colored part of your eye called?”), the remaining 12 facts were learned the previous week (6 from each source). Children were told that they had learned some of the questions the week before from the videos, but some facts they might have learned outside the laboratory (e.g., from a teacher or parent), and some facts they may not know. Each list of 22 facts had two random presentation orders, and these orders were counterbalanced across participants.

After each question was asked (e.g., “What is a group of rhinos called?”), the child was given the opportunity to answer freely. If the child indicated they did not know the answer, they were given four pre-determined multiple choice options (e.g., Mob, Crash, Herd, or School). Once the child had given an answer, the experimenter asked where the child had learned the information. Provided in the instructions at the beginning of the task were five example responses: parent, teacher, person in the video, puppet in the video, or they just knew. For each question, children were given the opportunity to answer the source memory question freely, but if they indicated they did not know where they had learned it, the five possible answers were provided.

Scores were calculated as proportion correct out of the total facts tested for both fact and source questions. For the present report, the primary variable of interest was the proportion of facts for which both the correct answer and correct source were identified (i.e., source memory conditionalized on fact memory). Six children did not complete the memory assessment.

#### Child Sleep Habits Questionnaire (CSHQ)

The CSHQ was used to provide a valid and reliable measure of habitual sleep time and habits (e.g., napping^35,36^). The 45-item questionnaire was completed by parents/guardians. For the present study, we examined responses to questions regarding the child’s usual amount of sleep each day (combining nighttime sleep and naps) and the child’s nap frequency (usually or 5–7 days, sometimes or 2–4 days, and rarely or 0–1 day). One additional parent of a 6-year-old child completed the CSHQ but reported a very low number of hours of sleep (i.e., 7 h per night). This value did not match the reported weeknight nor weekend wake and bedtimes. Given the inconsistencies in the questionnaire for this individual, their data was excluded from all analyses.

#### Verbal and spatial IQ

Estimates of intelligence were obtained using subtests from age-appropriate standardized intelligence tests (i.e., Wechsler Intelligence Scale for Children-Fourth Edition, or WISC, and the Wechsler Preschool and Primary Scale of Intelligence, or WPPSI). Scaled scores (SS) from the block design subtest, which reflects visual-spatial intelligence, and vocabulary, which reflects verbal intelligence, were obtained. One child was not administered the intelligence test due to lack of time.

#### Hippocampal subfield volumes

All participants participated in a mock scan prior to data collection. Ultra-high resolution structural scans were obtained of medial temporal lobe with a T2-weighted fast spin echo sequence (TR = 4120 ms, TE = 41 ms, 24 slices, 149 degree flip angle, voxel size 0.4 mm × 0.4 mm × 2 mm). Data were not included if children were (1) unable to enter the scanner, (2) unable to complete the scan, or (3) yielded data with too much motion to determine the anatomical boundaries required for segmentation. Results from this sample have been reported elsewhere^26^. Briefly, hippocampal subfield volumes were identified in the head and body of the hippocampus bilaterally. Consistent with previous literature, subfield volumes were not derived for the hippocampal tail due to its small size and the difficulty of accurately identifying subfield boundaries. Although there is disagreement regarding the ability to segment subfield boundaries in the hippocampal head using MRI, the current protocol focused on three large ROIs, collapsing across smaller subfields that tend to be more problematic. Moreover, Dice Similarity Coefficients (DSC) were calculated separately for head and body to ensure adequate reliability of the assessments. In both the head and body, three subfields were identified: subiculum, CA1, and a combination region of CA2–4/dentate gyrus (CA2–4/DG). The latter region combines multiple subfields, however, it includes both of the “late” developing subfields (CA3 and DG) and CA2, which is relatively small in size. Boundaries were adapted from La Joie et al.^38^ and are reported in detail elsewhere^26^.

Two raters blinded to the identity and age of the subjects independently traced 10 cases (2 from each of the age groups, e.g., 2 4-year-olds, 2 5-year-olds, etc.) bilaterally. DSCs were calculated to determine overlap and were as follows for each subregion and subfield: subiculum-head = 0.75, subiculum-body = 0.73, CA1-head = 0.72, CA1-body = 0.78, CA2–4/DG-head = 0.82, CA2–4/DG-body = 0.85. Intra-rater reliability was also assessed; DSCs were follows: subiculum-head = 0.75, subiculum-body = 0.73, CA1-head = 0.70, CA1-body = 0.78, CA2–4/DG-head = 0.81, CA2–4/DG-body = 0.87. DSC values above 0.7 are typically considered acceptable for agreement^39^; as such, overlap between the two raters indicated agreement.

One of the experienced raters then traced an additional 10 cases (again, 2 from each age group). These segmentations were combined with the 10 cases used for manual reliability (i.e., 20 total) and input into Automatic Segmentation of Hippocampal Subfields software (ASHS, Yushkevich et al.^40^). This yielded a study-specific template, which was subsequently used to generate hippocampal subfield volumes for the entire sample. All resulting segmentations were checked manually for quality. Segmentations with clear errors were omitted from further analysis (n = 8). No manual edits were made on the remaining segmentations, but it was noted that variability was greater in the head than the body due to greater variability of the underlying neuroanatomy of this region^41^.

In order to ensure that any observed effects were not the result of differences in brain size, subregion and subfield volumes were adjusted to control for differences in intracranial volume (ICV) using an analysis of covariance approach^42,43^. ICV values were calculated via Freesurfer and used to adjust volumes for variations in ICV for each age group, see^26,44^ for rationale. Note, analyses were conducted with raw volumes to ensure the ICV-correction did not drive any observed effects. These results were similar to results with ICV-adjusted volumes (see Supplemental Material).

### Analytic plan

Preliminary analyses revealed age (but not sex) was related to average 24-h sleep duration, r(184) = − 0.350, *p* = 0.000001. In addition, age (but not sex) was related to memory, r(193) = 0.619, *p* = 8.395E−22. Age and sex were related to hippocampal subfield volumes, as previously reported; for details see^26^.

Primary analyses examined relations between sleep and (1) memory and (2) hippocampal subfield volumes using partial correlation analyses, controlling for age and sex (Supplemental Table S1). Then, given previous findings of different patterns of results for brain–behavior relations in younger versus older children, analyses were conducted again for younger versus older children separately, via a median age split for children included in the analysis. Finally, effects of nap status (Table 2) were explored using univariate GLMs, again controlling for age and sex. Nappers consisted of children who napped 2 or more days per week (combining the “usually” and “sometimes” responses from the CSHQ) and non-nappers consisted of children who napped 0–1 times per week (“rarely” on the CSHQ). These analyses were conducted first in the younger age group, as there were very few (n = 5) nappers in the older age group.

**Table 2:** Descriptive statistics for 24-h sleep duration, nap status from the Child Sleep Habits Questionnaire (CSHQ) for 184 participants (104 younger, 80 older), and percent correct on the source memory task for 193 participants (111 younger, 80 younger).

	All	Younger	Older
Age mean years (SD)	6.19 (1.52)	5.04 (.76)	7.68 (.78)
24-h sleep duration (mins)	450–780	450–780	480–690
24-h sleep duration mean (SD)	609.51 (52.44)	622.07 (55.74)	593.19 (42.94)
**Nap status (n)**			
Napper (“Usually”, “Sometimes”)	58 (12,36)	43 (12,31)	5 (0,5)
Non-napper (“Rarely”)	130	60	70
Not reported	6	1	5
Source memory mean percent correct (SD)	22.82% (18.45)	14.26% (15.24)	34.40% (15.97)

## Supplementary information

Supplementary information.

## Data Availability

The data from the current study are available from the corresponding author on reasonable request.
