# Supplementary material for: Habitual sleep is associated with both source memory and hippocampal subfield volume during early childhood
Source: Sci Rep. 2020 Sep 17;10:15304. doi: 10.1038/s41598-020-72231-z (PMC7499159; doi:10.1038/s41598-020-72231-z)
Supplement: Supplementary file 1 — Supplementary information. [file 41598_2020_72231_MOESM1_ESM.docx]

**Supplemental Material**

Habitual sleep is associated with both source memory and hippocampal subfield

volume during early childhood

Tracy Riggins^1^* & Rebecca M. C. Spencer^2,3^

^1^Department of Psychology, University of Maryland, College Park

^2^Department of Psychological & Brain Sciences, University of Massachusetts, Amherst

^3^Institute for Applied Life Sciences, University of Massachusetts, Amherst

*Corresponding Author

Tracy Riggins

Department of Psychology

University of Maryland College Park

4094 Campus Dr.

College Park, MD 20742

301-405-5905

[riggins@umd.edu](mailto:riggins@umd.edu)

Supplemental Table 1. Bivariate correlations between age, sex, 24-hour sleep, memory, IQ, hippocampal volumes, and global brain volumes (*n*s = 137-199).

| *Variables* | | | 1 | 2 | 3 | 4 | 5 | 6 | 7 | 8 | 9 | 10 | 11 | 12 | 13 | 14 | 15 |
| --- | --- | --- | --- | --- | --- | --- | --- | --- | --- | --- | --- | --- | --- | --- | --- | --- | --- |
| 1 | Age |  | 1 |  |  |  |  |  |  |  |  |  |  |  |  |  |  |
| 2 | Sex | | -0.114 | 1 |  |  |  |  |  |  |  |  |  |  |  |  |  |
| 3 | 24-hour sleep duration |  | -0.350 | -0.056 | 1 |  |  |  |  |  |  |  |  |  |  |  |  |
| *Cognitive Measures* | | |  |  |  |  |  |  |  |  |  |  |  |  |  |  |  |
| 4 | Source memory | | 0.619 | -0.061 | -0.097 | 1 |  |  |  |  |  |  |  |  |  |  |  |
| 5 | Block design | | 0.151 | 0.015 | 0.099 | 0.073 | 1 |  |  |  |  |  |  |  |  |  |  |
| 6 | Vocabulary |  | 0.185 | -0.076 | 0.086 | 0.148 | 0.197 | 1 |  |  |  |  |  |  |  |  |  |
| *Hippocampal subfield volumes* | | |  |  |  |  |  |  |  |  |  |  |  |  |  |  |  |
| 7 | Head - Subiculum | | 0.032 | 0.310 | 0.041 | 0.004 | -0.086 | -0.011 | 1 |  |  |  |  |  |  |  |  |
| 8 | Head - CA1 | | 0.118 | 0.305 | -0.034 | 0.066 | -0.152 | -0.142 | 0.619 | 1 |  |  |  |  |  |  |  |
| 9 | Head - DG/CA2-4 | | 0.030 | 0.385 | 0.103 | 0.011 | -0.086 | -0.082 | 0.620 | 0.566 | 1 |  |  |  |  |  |  |
| 10 | Body - Subiculum | | 0.099 | -0.098 | -0.079 | 0.133 | 0.113 | 0.000 | 0.036 | 0.021 | -0.328 | 1 |  |  |  |  |  |
| 11 | Body - CA1 | | 0.168 | 0.101 | -0.152 | 0.048 | -0.066 | 0.005 | 0.194 | 0.500 | -0.064 | 0.333 | 1 |  |  |  |  |
| 12 | Body - DG/CA2-4 | | 0.207 | 0.033 | -0.188 | 0.225 | -0.069 | 0.027 | 0.026 | 0.278 | -0.200 | 0.416 | 0.738 | 1 |  |  |  |
| *Global brain volumes* | | |  |  |  |  |  |  |  |  |  |  |  |  |  |  |  |
| 13 | Total Gray Matter | | 0.187 | 0.281 | -0.043 | 0.169 | 0.232 | -0.041 | 0.169 | 0.038 | -0.047 | 0.134 | -0.015 | 0.054 | 1 |  |  |
| 14 | Subcortical Gray Matter | | 0.305 | 0.297 | -0.079 | 0.189 | 0.158 | 0.041 | 0.176 | 0.003 | 0.044 | 0.021 | -0.004 | 0.101 | 0.722 | 1 |  |
| 15 | Intracranial Volume |  | 0.294 | 0.375 | -0.111 | 0.228 | 0.320 | -0.027 | 0.066 | -0.037 | -0.105 | 0.143 | -0.030 | 0.035 | 0.853 | 0.640 | 1 |

Supplemental analyses with raw hippocampal volumes.

To ensure any observed effects were not the result of ICV-adjustment, analyses resulting in significant findings were re-conducted using raw volumes. In younger children, there remained a positive association between habitual 24-sleep duration and saw volume of CA2-4/DG in the head of the hippocampus, r(63) = .338, *p* = .005. Longer sleep durations were associated with larger subfield volumes. Raw volumes of CA1 in the body of the hippocampus also differed as a function of nap status within the younger age group, *F*(1,63) = 4.840, *p* = .031, ηp^2^ = .071. Volumes in nappers were larger than volumes in non-nappers.
